# Effect of Oxidized Low-Density Lipoprotein on Head and Neck Squamous Cell Carcinomas

**DOI:** 10.3390/biomedicines9050513

**Published:** 2021-05-05

**Authors:** Nadège Kindt, Fabrice Journé, Stéphane Carlier, Anne Trelcat, Alessandro Scalia, Sven Saussez

**Affiliations:** 1 Department of Clinical and Experimental Oncology, Institut Jules Bordet, Université Libre de Bruxelles, 1000 Brussels, Belgium; fabrice.journe@umons.ac.be; 2 Department of Human Anatomy and Experimental Oncology, UMONS Research Institute for Health Sciences and Technology, University of Mons (UMons), 7000 Mons, Belgium; anne.trelcat@umons.ac.be (A.T.); sven.saussez@umons.ac.be (S.S.); 3 Department of Cardiology, UMONS Research Institute for Health Sciences and Technology, University of Mons (UMons), 7000 Mons, Belgium; Stephane.carlier@umons.ac.be (S.C.); alessandro.scalia@umons.ac.be (A.S.); 4 Department of Cardiology, Centre Hospitalier Universitaire et Psychiatrique de Mons-Borinage, 7000 Mons, Belgium; 5 Department of Otorhinolaryngology and Head and Neck Surgery, CHU de Bruxelles, CHU Saint-Pierre, School of Medicine, Université Libre de Bruxelles, 1000 Brussels, Belgium

**Keywords:** HNSCC, oxLDL, Lox-1, cell migration, CD36

## Abstract

Cardiovascular disease (CVD) and cancer are two major causes of death worldwide. The question is, “Could there be a link between these two pathologies in addition to their shared, common risk factors?” To find some answers, we studied the effect of oxidized low-density lipoproteins (oxLDL) on head and neck cancer (HNC) cell lines, since oxLDL is a major contributor to atherosclerosis and the principal cause of CVD. In this study, we exposed three HNC cell lines (Detroit 562, UPCI-SCC-131 and FaDu) to oxLDL. We investigated two oxLDL receptors, CD36 and Lox-1, using immunofluorescence. Cancer cell migration was evaluated using Boyden chambers and the Wnt/β-catenin pathway was investigated using Western blotting. We demonstrated that the expression of CD36 and Lox-1 significantly increases after exposure to oxLDL. Moreover, we found that oxLDL reduces the migration of HNC cell lines, an observation that is in line with an increased degradation of β-catenin under oxLDL. Finally, the inhibition of CD36 with sulfosuccinimidyl oleate (SSO) reverses the inhibition of cell migration. In conclusion, we report that oxLDL seems to induce an increase in CD36 expression on HNC cell lines, enhancing the uptake of these lipids in cells to finally decrease cancer cell migration via the CD36/β-catenin pathway.

## 1. Introduction

Cardiovascular disease (CVD) and cancer are the two main causes of death worldwide. Indeed, the World Health Organization (WHO) has estimated that the global burden of cancer rose to 18.1 million new cases and 9.6 million deaths in 2018, with an estimated 5-year prevalence of 43.8 million, placing cancer as the second leading cause of death worldwide [1]. In parallel, CVD is the first leading cause of death globally, taking an estimated 17.9 million lives each year [2]. They share common risk factors, and their progression depends on oxidative stress, angiogenesis and inflammatory processes [3].

Oxidative stress occurs as a result of an increased production of reactive oxygen species (ROS) that promote the development of inflammatory processes in different tissues and, therefore, is relevant in the development of both atherosclerosis and cancer [3]. Atherosclerosis is the principal cause of CVD, defined as a chronic and progressive inflammatory state due to an immune response and an uncontrolled proliferation of vascular smooth muscle cells leading to the production of pro-inflammatory cytokines [3]. Activated endothelial cells express adhesion molecules on their surface such as vascular cell adhesion molecule-1 (VCAM-1) that bind leucocyte ligands and therefore promote leucocyte adhesion and transendothelial migration [4]. This inflammatory state is mainly caused by oxidized low-density lipoproteins (oxLDLs) that activate inflammasomes to produce IL-1β in endothelial cells, monocytes/macrophages and T cells [3]. Indeed, oxidized LDL is taken up by macrophages via scavenger receptors, such as lectin-like oxidized low-density lipoprotein receptor-1 (Lox-1) and CD36, which contribute to the formation of foam cells, thus leading to the progression of atherosclerotic vulnerable plaque [5]. 

In cancer, there is also a chronic and progressive inflammatory state where inflammation occurs due to the infiltration of immune cells such as macrophages, T cells and natural killer cells that release large amounts of inflammatory cytokines, pro-angiogenic factors and reactive oxygen species in the tumor microenvironment [3]. Monocytes/macrophages play a major role in both atherosclerosis and cancer. It was observed in head and neck squamous cell carcinomas that the infiltration of macrophages increased during tumor progression and is associated with a poor prognosis [6]. Furthermore, a specific population of macrophages originating from circulating monocytes called tumor-associated macrophages (TAMs) contribute to cancer progression and facilitate angiogenesis with their anti-inflammatory/pro-tumoral M2-like phenotype [7]. Interestingly, it was suggested by Wu et al. that unsaturated fatty acids polarize bone marrow-derived myeloid cells into an M2-like phenotype with a robust suppressive capacity [8]. Their investigation in colorectal cancer patients revealed an increase in lipid droplets in CD68^+^CD206^+^ (M2 markers) tumor-infiltrating myeloid cells when compared with the adjacent non-tumor tissue [8]. 

The consequences of atherosclerosis on cancer progression are still unknown. However, a recent study by Suzuki et al. has shown that atherosclerotic patients have a higher probability of developing cancer [9]. Indeed, the presence of coronary or aortic atherosclerosis is associated with a significantly higher risk of breast and colorectal cancers and lymphoma development. Moreover, a higher risk of these cancers is seen when atherosclerosis is found in multiple locations (coronary, aortic and peripheral artery diseases) [9]. Others have hypothesized that cancer treatments such as anthracycline-based chemotherapy in breast cancer and immune checkpoint inhibitors can induce atherosclerosis by notably increasing the level of metalloproteinases such as MMP-2 and MMP-9 [10,11]. Moreover, patients presenting chronic myeloid leukemia that were treated with the tyrosine kinase inhibitor ponatinib developed cardiovascular thromboembolism in 16% of cases [12]. In this context, patients with a high risk of atherosclerosis should be considered for treatment with aspirin and statins when they receive anti-cancer therapies and continuous surveillance should be performed for longer periods of time [13]. 

In this study, we have investigated whether oxLDL, largely involved in atherosclerosis progression, impacts the expression of two oxLDL receptors, CD36 and Lox-1, in head and neck cancer (HNC) cell lines. We sought to characterize the influence of oxLDL on cell migration and the involvement of the CD36 receptor in this migration.

## 2. Materials and Methods

### 2.1. Cell Culture

UPCI-SCC-131 and Detroit 562 (DSMZ, Braunschweig, Germany) cell lines were grown in Minimum Essential Medium (MEM, Gibco Life Technologies, Paisley, UK) and supplemented with 10% fetal bovine serum, 2 mM L-glutamine, 1% penicillin/streptomycin and 1% non-essential amino acids (Gibco Life Technologies, Paisley, UK). The FaDu cell line was grown in Dulbecco’s Modified Eagle Medium (DMEM, Lonza, Verviers, Belgium) and supplemented with 10% FBS, 2% L-glutamine and 1% penicillin/streptomycin. Routine cell culture was carried out at 37 °C in a humidified cell incubator at 5% CO_2_. All HNC cell lines used in this study had an HPV-negative status.

### 2.2. Oil Red O Staining

Human HNC cell lines were plated at a density of 30,000 cells/mL on sterile round glass coverslips in a 12-well dish. The following day, the medium was replaced with a fresh serum-free medium, with or without 30 µg oxLDL. After 48 h of exposure, cell monolayers were fixed with 4% paraformaldehyde in Dulbecco’s Phosphate-Buffered Saline (DPBS, Lonza, Verviers, Belgium). Cells were rinsed with DPBS and colored with Oil Red O (Merk Sigma, Darmstadt, Germany) for 15 min at room temperature. Cells were rinsed three times with distilled water and the slides were mounted with aquatex^®^ (Merk Sigma, Darmstadt, Germany). The appearance of cells, with or without oxLDL, was documented by phase-contrast microscopy using a Zeiss axioplan microscope equipped with a color charge-coupled device (CCD) camera (ProgRes C10plus, Jenoptik, Jena, Germany).

### 2.3. Immunofluorescence Microscopy

HNC cells were plated at a density of 30,000 cells/mL on sterile round glass coverslips in a 12-well dish. The following day, the medium was replaced with a fresh serum-free medium, with or without 30 µg oxLDL. After 48 h of exposure, cell monolayers were fixed with 4% paraformaldehyde in DPBS. Before the application of antibodies, the cell monolayers were rinsed several times with PBS (0.04 M Na_2_HPO_4_, 0.01 M KH_2_PO_4_, 0.12 M NaCl, pH 7.2) containing 0.1% Triton X-100 (the same detergent-containing buffer was used for subsequent incubations and rinsing steps). Before exposure to the primary antibodies, the cells were preincubated for 20 min in PBS containing 0.05% casein to prevent non-specific adsorption of immunoglobulins. Cells were exposed overnight to the primary antibody (either anti-CD36 (Merk Sigma, Darmstadt, Germany) or anti-Lox-1 (ThermoFisher Scientific, Waltham, MA, USA)), which had been diluted at 1:200 and 1:1000, respectively, in PBS containing 0.05% casein. On the following day, the cells were exposed to an anti-rabbit IgG antibody coupled with Alexa 594 for 30 min (ThermoFisher Scientific, Waltham, MA, USA). After the final rinses in PBS, the coverslips were mounted onto glass slides using a commercial anti-fading medium (Vectashield, Vector Laboratories, Burlingame, CA, USA). Confocal microscopy observations were carried out using an Olympus FV1000D laser scanning inverted microscope equipped with a red laser diode (LD559) (Olympus, Tokyo, Japan).

### 2.4. Cell Migration Assay

Cell migration was assessed using a Boyden chamber assay consisting of 24-well plates (lower chambers) with cell culture inserts (upper chambers), where both chambers were separated using a polycarbonate membrane (8 µm size) (ThermoFisher Scientific, Waltham, MA, USA). HNC cells were seeded in cell culture inserts (5 × 10^5^ cells/insert) in a serum-free medium (DMEM). The lower chambers were filled with a complete medium (DMEM, 10% FBS) with or without 30 µg oxLDL. After 96 h, the cells were wiped from the upper surface of the cell culture inserts with a cotton-tipped swab and the migrating cells were stained with crystal violet. Five microscopic fields (×10 magnification) were taken using a Zeiss Axio scope A1 microscope (Carl Zeiss, Oberkochen, Germany). The surface area covered by cells was calculated using ImageJ software (a public domain image software developed by W. Rasband at the Research Services Branch of the National Institute of Health, NIH, Bethesda, MD, USA) [14]. 

For the inhibition of CD36 with sulfosuccinimidyl oleate (SSO), an irreversible inhibitor of CD36 that leads to a reduction in fatty acid uptake [15], Detroit 562 cells were seeded in cell culture inserts (6.5 × 10^5^ cells/insert) in a serum-free medium (DMEM). On the next day, the cells were treated with 1µM of SSO (Cayman chemical, Ann Arbor, MI, USA) or were SSO free for 4 h in a serum-free medium. After 4 h, cells were rinsed with DPBS and a serum-free medium was added to the insert. The lower chambers were filled with a complete medium (DMEM, 10% FBS) with or without 30 µg oxLDL. After 72 h, cells were stained with crystal violet. The surface area covered by cells was calculated using ImageJ software [14].

### 2.5. Western Blotting

At 80% confluency in Petri dishes, human HNC cell lines were treated with or without 30 µg oxLDL for 6 h. After oxLDL exposure, the cells were lysed using a detergent solution (RIPA buffer) supplemented with protease and phosphatase inhibitors (all reagents from Pierce, ThermoFisher Scientific, Waltham, MA, USA). Protein concentrations were determined by a BCA protein assay (Pierce, ThermoFisher Scientific, Waltham, MA, USA) using bovine serum albumin as the standard. Extracted proteins (30 μg) were loaded on 4–20% Mini-PROTEAN TGX gels (SDS) (Bio-Rad Laboratories, München, Germany) and separated using electrophoresis. Then, proteins in the gel were electrotransferred onto nitrocellulose membranes (iBlot^®^ Dry Blotting System, Life Technologies-Invitrogen, Ghent, Belgium). Immunodetection was performed using an anti-β-catenin antibody (1/1000), an anti-p-β-catenin antibody (to detect the phosphorylation of β-catenin at the S675; 1/1000) (Cell Signaling, Danvers, MA, USA) and an anti-actin antibody (1/1000) (Pierce, ThermoFisher Scientific, Waltham, MA, USA). A peroxidase-labeled anti-rabbit IgG antibody (1/5000) (Amersham Pharmacia Biotech, Roosendaal, the Netherlands) and a peroxidase-labeled anti-mouse IgG antibody (1/2000) were used as the secondary antibodies. Bound peroxidase activity was detected using the SuperSignal^®^ West Pico Chemiluminescent Substrate (Pierce, ThermoFisher Scientific, Waltham, MA, USA) following the manufacturer’s instructions. The bands were visualized by exposing the membranes to photosensitive film (Hyperfilm ECL, Amersham Pharmacia Biotech, Roosendaal, the Netherlands). Band intensities were quantified using ImageJ software [14] and a p-β-catenin/β-catenin relative ratio was calculated by dividing the mean intensity of each band. Then, fold change was calculated using these ratios to compare the treated and untreated cells.

### 2.6. Statistical Analysis

SigmaPlot^®^ version 11 software (Systat Software, San Jose, CA, USA) was used for the statistical analyses. Parametric analyses were achieved using the Student’s *t*-test, after checking for normality using the Shapiro–Wilk test. Data are expressed as means ± SD with a *p* ≤ 0.05 value to indicate a statistically significant difference.

## 3. Results

### 3.1. OxLDL Increases the Expression of CD36 and Lox-1 in Head and Neck Cancer Cell Lines

**Figure 1:**
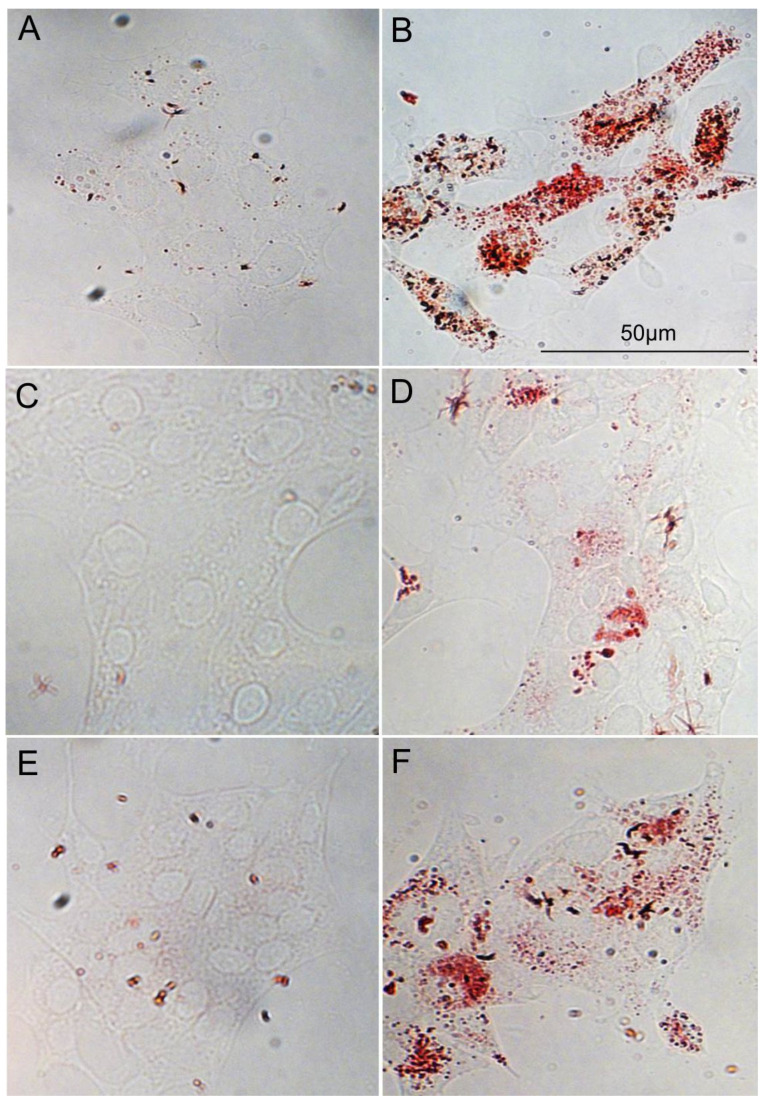
Uptake of oxLDL by HNC cells. (**A**,**B**) Representative pictures of Oil Red O staining of FaDu cells without oxLDL and with oxLDL, (**C**,**D**) Detroit 562 cells without oxLDL and with oxLDL and (**E**,**F**) UPCI-SCC-131 cells without oxLDL and with oxLDL. Cells were colored with Oil Red O after 48 h of oxLDL exposure.

**Figure 2:**
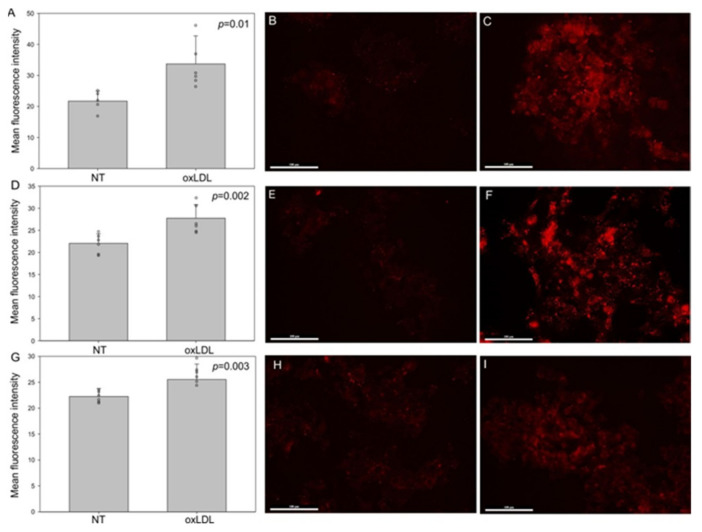
CD36 expression in HNC cell lines. (**A**,**D**,**G**) Mean fluorescence intensity of CD36 increased significantly under oxLDL exposure in FaDu cells (*t*-test, *p* = 0.01, *n* = 6), Detroit 562 cells (*t*-test, *p* = 0.002, *n* = 6) and UPCI-SCC-131 cells (*t*-test, *p* = 0.003, *n* = 6) (mean ± SD; o: datapoint). (**B**,**E**,**H**) Representative immunofluorescence of CD36 in FaDu, Detroit 562 and UPCI-SCC-131 cells, respectively, without oxLDL (NT) or (**C**,**F**,**I**) treated with 30 µg of oxLDL.

**Figure 3:**
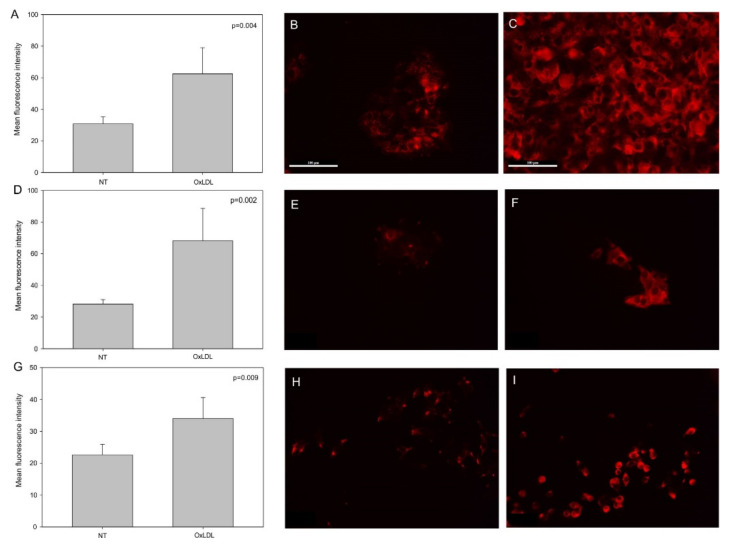
Lox-1 expression in HNC cell lines. (**A**,**D**,**G**) Mean fluorescence intensity of Lox-1 increased significantly under oxLDL exposure in FaDu cells (*t*-test, *p* = 0.004, *n* = 6), Detroit 562 cells (*t*-test, *p* = 0.002, *n* = 6) and UPCI-SCC-131 cells (*t*-test, *p* = 0.009, *n* = 6) (mean ± SD, o: datapoint). (**B**,**E**,**H**) Representative immunofluorescence of Lox-1 in FaDu, Detroit 562 and UPCI-SCC-131 cells, respectively, without oxLDL (NT) or (**C**,**F**,**I**) with 30 µg of oxLDL.

First, we determined that oxLDL can be internalized by all of our HNC cells, as demonstrated by the Oil Red O staining (Figure 1). Indeed, Oil Red O staining allowed us to visualize the lipids taken up by cells after 48 h of oxLDL exposure (Figure 1B,D,F). Moreover, CD36 and Lox-1 expression significantly increased after oxLDL exposure in the three HNC cell lines, as shown by immunofluorescence (Figure 2A (CD36 mean fluorescence intensity [MFI] in FaDu cells: 21 [NT] vs. 33 [oxLDL], *t*-test, *p* = 0.01), -D (CD36 MFI in Detroit 562 cells: 22 [NT] vs. 28 [oxLDL], *t*-test, *p* = 0.002), -G (CD36 MFI in UPCI-SCC131 cells: 22 [NT] vs. 25.5 [oxLDL], *t*-test, *p* = 0.003) and Figure 3A (Lox-1 MFI in FaDu cells: 31 [NT] vs. 62 [oxLDL], *t*-test, *p* = 0.004), -D (Lox-1 MFI in Detroit 562 cells: 28 [NT] vs. 68 [oxLDL], *t*-test, *p* = 0.002), -G (Lox-1 MFI in UPCI-SCC131 cells: 22 [NT] vs. 34 [oxLDL], *t*-test, *p* = 0.009), respectively). Figure 2 and Figure 3C,F,I illustrate an increased fluorescence intensity, demonstrating a rise in CD36 and Lox-1 expression, respectively. These results can explain the internalization of oxLDL in cancer cells.

### 3.2. Cell Migration Decreases and β-Catenin Phosphorylation Increases after oxLDL Exposure

**Figure 4:**
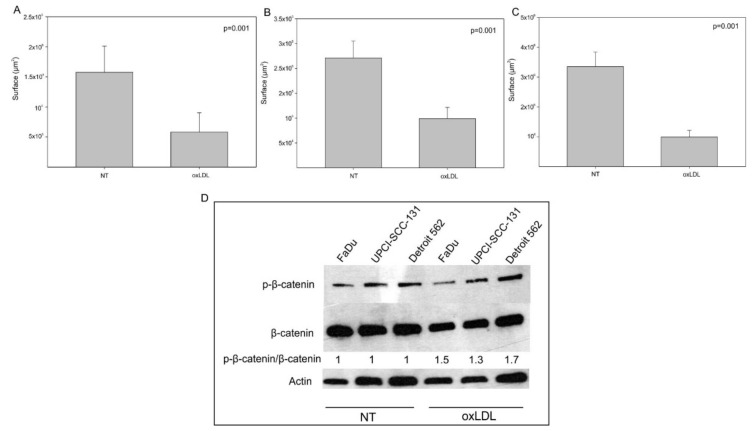
Decreased migration of HNC cell lines under oxLDL exposure. (**A**–**C**) Surface area covered by cells decreased significantly after oxLDL exposure in FaDu cells (*t*-test, *p* = 0.001, *n* = 6), Detroit 562 cells (*t*-test, *p* = 0.001, *n* = 6) and UPCI-SCC-131 cells (*t*-test, *p* = 0.001, *n* = 6), respectively (mean ± SD; o: datapoint) (NT: cells without oxLDL). (**D**) Representative Western blotting of p-β-catenin and β-catenin in HNC cell lines exposed or not exposed to oxLDL for 6 h. Actin is presented as the loading control. Fold change of p-β-catenin in untreated and treated cells.

In order to assess whether oxLDL impacts HNC cell migration, we used Boyden chambers and showed that oxLDL decreased cell migration in all HNC cell lines (Figure 4A (surface area covered by FaDu cells: 1.57 × 10^6^ µm^2^ [NT] vs. 0.58 × 10^6^ µm^2^ [oxLDL], *t*-test, *p* = 0.001), -B (surface area covered by Detroit 562 cells: 2.2 × 10^5^ µm^2^ [NT] vs. 0.99 × 10^5^ µm^2^ [oxLDL], *t*-test, *p* = 0.001), -C (surface area covered by UPCI-SCC131 cells: 3.3 × 10^6^ µm^2^ [NT] vs. 0.99 × 10^6^ µm^2^ [oxLDL], *t*-test, *p* = 0.001)). To understand this result, we explored the Wnt/β-catenin pathway, which is involved in cell motility and migration [16]. Western blotting demonstrated that the p-β-catenin/β-catenin fold change increases under oxLDL exposure compared to non-treated cells (Figure 4D), especially in FaDu and Detroit 562 cells, where the fold change was 1.5 and 1.7, respectively. This result seems to confirm the decrease in cell migration after treatment with oxLDL. 

### 3.3. Involvement of CD36 in Cell Migration under oxLDL Exposure

**Figure 5:**
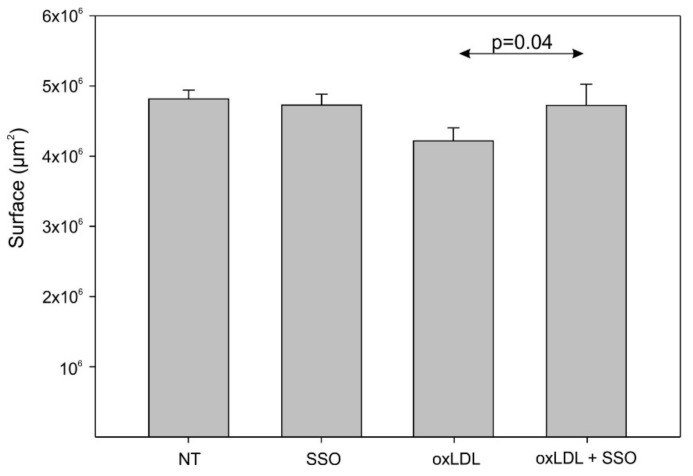
Detroit 562 cells’ migration under oxLDL and SSO treatment. There is no significant difference between the control cells (NT) and the SSO-treated cells, but SSO significantly overcomes the effect of oxLDL on cell migration (*t*-test, *p* = 0.04, *n* = 6) (mean ± SD; o: datapoint) (NT: cells without oxLDL).

To investigate the mechanisms involved in the cell migration decline after oxLDL exposure, we explored the implication of CD36 in Detroit 562 cells after its inhibition with 1 µM of SSO. No change in cell migration appeared between the control cells (NT) and the SSO-treated cells, but oxLDL exposure led to a decrease in cell migration that was significantly overcome by the SSO treatment (Figure 5, surface area covered by cells: 4.2 × 10^6^ µm^2^ (oxLDL) vs. 4.7 × 10^6^ µm^2^ (oxLDL + SSO), *t*-test, *p* = 0.04). The involvement of CD36 in the decreasing cell migration induced by oxLDL is reflected in the significant increase in the surface area covered by cells under oxLDL and SSO treatment.

## 4. Discussion

The mechanisms that could link atherosclerosis and cancer development are not known, but an explanation might be the dysregulation of lipid metabolism and, notably, the increased production of oxLDL as a result of elevated oxidative stress [17]. More precisely, during inflammation or the dysregulation of lipid metabolism, the production of ROS increases, leading to an oxidative modification of LDL that is converted into oxLDL [17]. Lipid metabolism is described as one of the most important metabolic pathways involved in many aspects of cancer cell function, including pathways related to cell transformation and tumor development [17]. Recently, several studies have explored the action of different types of lipids and/or related pathways in cancer progression, malignancy and metastasis [18,19,20]. Some studies have indicated an association between obesity, metabolic syndrome and aggressiveness of tumor growth [21,22,23]. Moreover, oxLDL is also linked to metabolic diseases such as diabetes and obesity, in which an important level of low-density lipoprotein (LDL) circulating in the blood can be oxidized [24]. 

Here, we have explored the effect of oxLDL, a major player in atherosclerosis, on HNC cell behavior. The first observation that we have made is that all HNC cell lines, with some differences in the internalization, take up oxLDL, as illustrated by Oil Red O staining. It was demonstrated that distinct mechanisms are involved in the oxLDL internalization by CD36 and scavenger receptor class B type 1 (SR-B1) (another oxLDL receptor) in macrophages, where the oxLDL uptake by CD36, but not by SR-BI, is dependent on dynamin [25]. However, in HNC cell lines, these oxLDL endocytic mechanisms are unknown. Some studies have already investigated oxLDL involvement in prostate, colorectal and lung cancer, which all demonstrated that oxLDL promotes cancer metastasis via Lox-1 activation in vitro and in vivo [26,27,28]. Notably, Gonzalez et al. demonstrated, in prostate cancer, a significant increase in Lox-1 expression in adenocarcinoma compared to normal prostate tissue [26]. They also observed that oxLDL induced epithelial-to-mesenchymal transition (EMT) through Lox-1 activation, shown by a lower expression of epithelial markers such as E-cadherin and plakoglobin and a higher level of mesenchymal markers such as vimentin and N-cadherin [26]. Likewise, in a metastasis colorectal mouse model, it was shown that the downregulation of Lox-1 by RNAi led to a reduction in the number and volume of metastatic tumors that were associated with a decrease in Ki67 and VEGF-A165 expression [27]. This is the first time that oxLDL has been explored in HNC, and it has been shown that Lox-1 expression increases after oxLDL exposure. However, we did not observe an increase in cell migration in vitro, maybe due to the fact that we did not expose the cancer cells directly to oxLDL in the Boyden chamber assay. Indeed, the oxLDL was placed in the lower chamber and not in the upper insert with the cells. We made this decision in order to mimic reality, where cancer cells are not in direct contact with oxLDL. 

Regarding CD36, we have demonstrated that its expression increases under oxLDL exposure, which is not demonstrated elsewhere in cancer. In ovarian and cervical cancer, it was observed that CD36, as Lox-1, leads to cancer progression and metastasis [29,30]. Indeed, the expression of CD36 increases in metastatic ovarian tumors compared to matched primary tumors [29]. Lidanyi et al. suggest that omental adipocytes reprogram tumor metabolism through the upregulation of CD36 in ovarian cancer cells [29]. Interestingly, a subpopulation of CD44^bright^ cells were described in oral cancer as initiating metastasis. They did not overexpress the mesenchymal genes, were slow cycling and expressed high levels of the fatty acid receptor CD36 and lipid metabolism genes [31]. They also showed that palmitic acid or a high-fat diet specifically raised the metastatic potential of CD36+ metastasis-initiating cells in a CD36-dependent manner. Moreover, the use of CD36-neutralizing antibodies caused almost complete inhibition of metastasis in several mouse models of human oral cancer, with no side effects [31]. However, the study of Fang et al. demonstrated, in vitro and in vivo, that CD36 repressed colorectal metastasis by stimulating the proteasome-dependent ubiquitination of glypican 4, followed by the inhibition of aerobic glycolysis through the arrest of β-catenin/c-myc signaling and the suppression of the glucose transporter 1 and lactate dehydrogenase A genes [32]. In fact, their colorectal mouse model exhibited no liver metastasis induction after the injection of colorectal cell line overexpressing CD36 compared to the control group [32]. This research supports our results, where we observed a reduction in cell migration associated with increased degradation of β-catenin under oxLDL exposure, keeping in mind that CD36 expression increases in the presence of oxLDL. Thus, we have conducted our research on CD36 and discovered that its inhibition with SSO leads to a rescue of cell migration under oxLDL exposure. 

**Figure 6:**
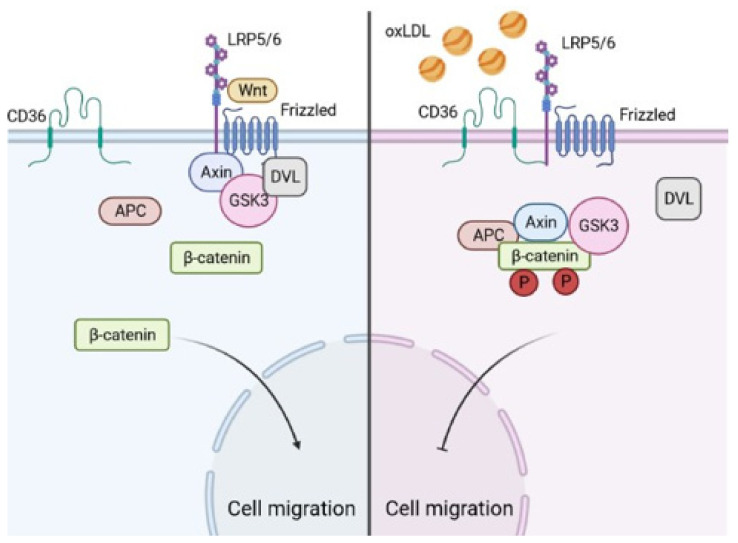
Schematic representation of cell migration inhibition by oxLDL. On the left: without oxLDL, Wnt ligands activate cell signaling by binding the Frizzled and LRP5/6 receptors at the cell surface, which leads to the delivery of β-catenin that induces cell migration. On the right: in the presence of oxLDL, CD36 interacts with the Frizzled and LRP5/6 receptors, bypassing Wnt, which leads to the degradation of β-catenin and results in a reduction in cell migration. Figure created using BioRender.com, accessed on 5 April 2021.

Regarding the Wnt/β-catenin pathway, it is well known that without the stimulation of this pathway, β-catenin is phosphorylated by the destruction complex, which is composed of adenomatous polyposis coli, Axin 1/2, casein kinase I and glycogen synthase kinase 3β [16]. Wnt ligands activate canonical signaling by binding Frizzled and LRP5/6 receptors at the cell surface, prompting the dissociation of the destruction complex and delivering β-catenin to the nucleus [16]. Interestingly, the co-receptor of Wnt is a low-density lipoprotein receptor-related protein (LRP), and, in another field of research, it was demonstrated in mesenchymal stem cells that under oxLDL stimulation, the expression of CD36 increases. More importantly, CD36 interacts with Frizzled and LRP5/6 receptors by bypassing Wnt, which leads to the destabilization and degradation of β-catenin, resulting in a reduction in osteoblast differentiation [33]. The important role of calcifications in atherosclerotic plaques has emerged lately as an additional risk factor for major acute coronary events, mainly through the presence of microcalcifications in the thin-cap fibroatheroma that might lead to plaque rupture [34,35]. Our results demonstrate that cell migration under oxLDL is recovered after CD36 inhibition. This could be explained by the fact that CD36 cannot interact with LRP5/6 and Frizzled receptors; indeed, the interaction between CD36 and LRP5/6/Frizzled led to the degradation of β-catenin, resulting in a reduction in cell migration (Figure 6). 

Another aspect of this study is the use of HPV-negative HNC cell lines, which represent the major HNC population. However, HPV infection is estimated to cause 22% of oropharyngeal cancer and 47% of tonsillar cancer cases [36]. A study by Al-Eitan et al. demonstrated that three miRNAs (miR-27b, miR-1914-3p and miR-612) were up- and downregulated in warts (skin lesion due to low-risk HPV) compared to normal skin and, more importantly, that these three miRNAs interacted with the *OLR1* gene coding for the Lox-1 receptor [37]. In this context, the effects of oxLDL on HPV-positive cells should be of great importance. 

In conclusion, oxLDL seems to induce an increased expression of both the Lox-1 and CD36 receptors on HNC cell lines, enhancing their uptake of oxidized LDL and decreasing cancer cell migration via the CD36/β-catenin pathway. However, more clarifications are needed to verify the exact contribution of CD36 and Lox-1 in cancer progression. Indeed, studies demonstrated that the upregulation of CD36 is associated either with tumor progression or tumor repression [29,32]. Finally, future experiments should be conducted on the involvement of oxLDL in cell migration and on the oxLDL receptors’ expression in HPV-positive HNC cell lines. Moreover, it will be interesting to analyze whether the expression of Lox-1 and CD36 could be associated with a prognostic value in HNC patients.

## Data Availability

Data are contained within the article.
